# Supplementary material for: Sex Differences in the Prevalence of Head and Neck Cancers: A 10-Year Follow-Up Study of 10 Million Healthy People
Source: Cancers (Basel). 2022 May 20;14(10):2521. doi: 10.3390/cancers14102521 (PMC9139445; doi:10.3390/cancers14102521)
Supplement: Supplementary file 1 [file cancers-14-02521-s001.zip › cancers-1699301-supplementary.pdf]

Table S1. The International Classification of Disease, Tenth Revision, Clinical Modification (ICD-10-CM) codes used to diagnose head-and-neck cancers.

The head-and-neck cancers	ICD-10 codes
Laryngeal cancer	C32.0, C32.1, C32.2, C32.3, C32.9, C32.8
Sino-nasal cancer	C31.0, C31.1, C31.2, C31.3, C31.8, C31.9
Hypopharyngeal cancer	C12, C13.0, C13.1, C13.2, C13.8, C13.9
Oropharyngeal cancer	C01, C02.4, C09.0, C09.1, C09.8, C09.9, C10.0, C10.1, C10.2, C10.3, C10.8, C10.9, C14.0, C14.2, C14.8
Oral cancer	C02.0, C02.1, C02.2, C02.3, C02.8, C02.9, C03.0, C03.1, C03.9, C04.9, C05.2, C05.8, C05.9, C06.0, C06.1, C06.2, C06.8, C06.9
Nasopharyngeal cancer	C11.0, C11.1, C11.2, C11.3, C11.8, C11.9
Salivary gland cancer	C07, C08.0, C08.1, C08.8, C08.9

Table S2. The incidence of head-and-neck cancers in healthy men and women by age during the 10-year follow-up period.

	Age group	Male			Female		
		IR	Lower	Upper	IR	Lower	Upper
Overall Head Neck Cancer	20-29	0.018	0.015	0.023	0.017	0.013	0.021
	30-39	0.041	0.037	0.045	0.027	0.023	0.033
	40-49	0.115	0.109	0.121	0.046	0.042	0.05
	50-59	0.296	0.285	0.308	0.066	0.06	0.071
	60-69	0.538	0.518	0.559	0.099	0.091	0.108
	70-	0.711	0.677	0.747	0.15	0.137	0.164
Laryngeal Cancer	20-29	0.001	0.0004	0.0024	0.0004	0.0001	0.0017
	30-39	0.0038	0.0028	0.0051	0.0005	0.0001	0.0019
	40-49	0.022	0.02	0.025	0.002	0.0013	0.0031
	50-59	0.1	0.093	0.107	0.0043	0.0031	0.0059
	60-69	0.212	0.2	0.225	0.0074	0.0054	0.01
	70-	0.299	0.277	0.322	0.014	0.011	0.019
Sino-nasal Cancer	20-29	0.0012	0.0005	0.0027	0.001	0.0004	0.0025
	30-39	0.0034	0.0025	0.0047	0.0012	0.0005	0.0028
	40-49	0.0052	0.004	0.0067	0.0036	0.0026	0.005
	50-59	0.012	0.0098	0.015	0.0055	0.0041	0.0073
	60-69	0.021	0.017	0.025	0.0077	0.0057	0.01
	70-	0.027	0.021	0.034	0.013	0.0094	0.017
Hypopharyngeal Cancer	20-29	0.0002	0	0.0014	0.0002	0	0.0015
	30-39	0.0007	0.0004	0.0014	0.0009	0.0004	0.0025
	40-49	0.0055	0.0043	0.0071	0.0008	0.0004	0.0016
	50-59	0.026	0.023	0.03	0.0019	0.0011	0.003
	60-69	0.071	0.064	0.079	0.0029	0.0018	0.0047
	70-	0.105	0.092	0.119	0.0048	0.0029	0.008
Oropharyngeal Cancer	20-29	0.002	0.0011	0.0038	0.001	0.0004	0.0025
	30-39	0.007	0.0056	0.0087	0.0024	0.0013	0.0044
	40-49	0.026	0.023	0.029	0.0051	0.0039	0.0067
	50-59	0.061	0.056	0.066	0.0099	0.008	0.012
	60-69	0.085	0.078	0.094	0.012	0.0098	0.016
	70-	0.109	0.096	0.123	0.016	0.012	0.021
Oral Cancer	20-29	0.0049	0.0033	0.0072	0.0056	0.0039	0.0082
	30-39	0.011	0.0089	0.013	0.011	0.0086	0.015
	40-49	0.024	0.021	0.027	0.014	0.012	0.017
	50-59	0.049	0.044	0.054	0.02	0.018	0.024
	60-69	0.078	0.071	0.086	0.037	0.032	0.043
	70-	0.104	0.091	0.118	0.064	0.056	0.074
Nasopharyngeal Cancer	20-29	0.004	0.0026	0.0063	0.0021	0.0011	0.0039
	30-39	0.0084	0.0069	0.01	0.0038	0.0023	0.0062
	40-49	0.022	0.019	0.024	0.0066	0.0052	0.0084
	50-59	0.028	0.025	0.032	0.0076	0.0059	0.0096
	60-69	0.036	0.031	0.042	0.011	0.0089	0.015

	70-	0.034	0.027	0.043	0.012	0.0089	0.017
Salivary gland Cancer	20-29	0.0051	0.0034	0.0075	0.0063	0.0044	0.009
	30-39	0.0071	0.0057	0.0089	0.0073	0.0052	0.01
	40-49	0.012	0.01	0.015	0.014	0.012	0.016
	50-59	0.023	0.02	0.027	0.017	0.014	0.02
	60-69	0.039	0.034	0.045	0.022	0.018	0.026
	70-	0.045	0.037	0.055	0.026	0.021	0.033
